# Hepatitis B Virus Genotype D: An Overview of Molecular Epidemiology, Evolutionary History, and Clinical Characteristics

**DOI:** 10.3390/microorganisms11051101

**Published:** 2023-04-22

**Authors:** Thaís B. Sant’Anna, Natalia M. Araujo

**Affiliations:** Laboratory of Molecular Virology and Parasitology, Oswaldo Cruz Institute, FIOCRUZ, Rio de Janeiro 21041-250, RJ, Brazil; thaisbfsantanna@gmail.com

**Keywords:** hepatitis B virus, genotype D, subgenotypes, geographic distribution, mutations, evolution

## Abstract

The hepatitis B virus (HBV) genotype D (HBV/D) is the most extensively distributed genotype worldwide with distinct molecular and epidemiological features. This report provides an up-to-date review on the history of HBV/D subgenotyping and misclassifications, along with large-scale analysis of over 1000 HBV/D complete genome sequences, with the aim of gaining a thorough understanding of the global prevalence and geographic distribution of HBV/D subgenotypes. We have additionally explored recent paleogenomic findings, which facilitated the detection of HBV/D genomes dating back to the late Iron Age and provided new perspectives on the origins of modern HBV/D strains. Finally, reports on distinct disease outcomes and responses to antiviral therapy among HBV/D subgenotypes are discussed, further highlighting the complexity of this genotype and the importance of HBV subgenotyping in the management and treatment of hepatitis B.

## 1. Introduction

Despite rapid medical advances that have led to the development of several generations of vaccines, improved screening tests, and effective treatment options, hepatitis B remains one of the most prevalent viral infections in humans and a major global public health problem. An estimated two billion people have serological evidence of past or present hepatitis B virus (HBV) infection and 1.5 million new infections are reported annually worldwide. In 2019, around 296 million chronic carriers of hepatitis B were documented, resulting in 820,000 deaths [1]. The natural history of HBV infection has a diverse and broad range of outcomes, ranging from acute, inactive to active chronic infection, which can progress to liver cirrhosis and hepatocellular carcinoma (HCC) [2]. There is presently no definitive cure for chronic HBV infection, although the virus can be effectively controlled and disease progression limited with nucleos(t)ide analogs (NA) and peginterferon (peg-IFN), which represent the mainstay of HBV therapy [3]. Complex interplay between host and virus genetics, together with environmental factors, contributes to the progression to severe liver disease (cirrhosis and HCC) in a subset of individuals, with variable global incidence rates [4,5]. In view of the considerable public health concern posed by HBV, comprehensive understanding of both viral and host characteristics is essential to facilitate eradication of HBV infection in the near future.

HBV is the prototype member of the family *Hepadnaviridae* containing a partially double-stranded circular DNA genome of ~3200 nucleotides (nt). The HBV genome has a highly compact coding structure consisting of four partly or completely overlapping reading frames, specifically, pre-S1/pre-S2/S that codes for the surface proteins S, M, and L of the HBs antigen (HBsAg), pre-C/C for e antigen (HBeAg) and core protein (HBcAg), P for polymerase/reverse transcriptase, and X for a transcriptional transactivator protein (HBxAg) [6]. Despite the severe constraints imposed by this genomic organization, the lack of proofreading activity of reverse transcriptase contributes to the emergence of significant diversity among HBV isolates [7]. Additionally, recombination is an important element of genetic variability, with several variants generated by recombination events between different HBV genotypes documented worldwide [8,9]. Based on >7.5% genomic sequence divergence, HBV has been phylogenetically classified into nine genotypes (A–I), with another putative genotype (J) isolated from a single individual [10]. The significant diversity within genotypes A–D, F, and I has led to further classification into subgenotypes with increased numbering over time. Several guidelines for HBV subgenotyping have been established: (i) intergroup nucleotide divergence of 4–7.5% over the full-length genome; (ii) monophyletic clustering with significant bootstrap support (>75%) in the phylogenetic tree; (iii) recombinant HBV sequences should not be classified as novel subgenotypes but reported separately; (iv) the novel subgenotype should have unique nucleotide and amino acid polymorphisms [11,12,13]. HBV (sub)genotypes differ significantly in terms of frequency of clinically relevant mutations (i.e., immune escape-, drug resistance-, and HCC-associated mutations) [14], contributing to differences in disease progression, response to antiviral therapy, and clinical outcomes [15,16,17,18]. Moreover, a clear geographic correlation in the global distribution of HBV genotypes, and, in some cases subgenotypes, has been reported, most likely reflecting historical patterns of human migration [19,20]. Remarkably, recent discoveries of HBV DNA from archaeological remains indicate HBV infection in the human population for millennia [19,21,22]. However, knowledge of the evolutionary processes involved in the emergence and dispersal of the major modern HBV genotypes is currently limited.

In this review, we provide a comprehensive summary of the most relevant aspects of HBV genotype D (HBV/D), including its subgenotyping history, temporal and geographic origins, and virological and clinical characteristics. Additionally, data from 1156 full-length genome sequences were collected with the aim of obtaining in-depth information on the prevalence and geographic distribution of HBV/D subgenotypes worldwide.

## 2. Genotype D

### 2.1. History of HBV/D Subgenotyping

HBV/D was one of the four original HBV genotypes (along with HBV/A, B, and C) defined in 1988 by Okamoto and colleagues, initially named as groups based on an intergroup divergence in a nucleotide sequence of 8% or higher from 18 complete genomes [23]. Compared to the other genotypes, HBV/D has the shortest genome (3182 nucleotides in length) and is characterized by a 33 nt deletion at the start of the pre-S1 region (at nt2854) that generates a truncated but viable L protein with a myristylation site and intact hepatocyte recognition site [7]. The classical precore (PC) mutation, located at the HBV nucleotide position 1896 and consisting of a G-A substitution that creates a stop codon preventing translation of the HBeAg precursor, has been frequently identified in HBV/D [24]. Consequently, in newborns/children infected with HBV/D, seroconversion from HBeAg to anti-HBe usually occurs during adolescence or early adulthood, indicating a greater tendency of horizontal rather than vertical transmission for this genotype.

**Figure 1:**
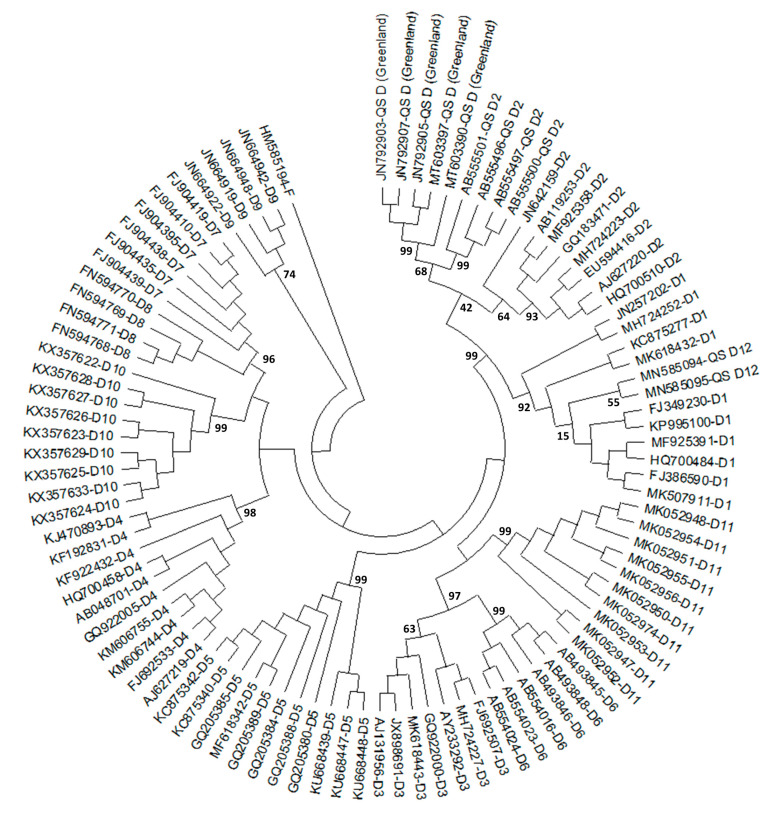
Phylogenetic tree of 96 hepatitis B virus (HBV) genotype D (HBV/D) complete genome sequences inferred by using the maximum likelihood method. Values at internal nodes indicate percentage of 500 bootstrap replicates that support the group. HBV reference sequences are indicated by their accession numbers followed by their subgenotype. QS, quasi-subgenotype.

To date, up to 11 HBV/D subgenotypes have been classified (Figure 1). Based on examination of 33 full-length HBV genomes, Norder and colleagues (2004) initially classified HBV/D into four subgenotypes, denoted as D1, D2, D3, and D4 [25]. In 2006, subgenotype D5 was assigned to two HBV strains detected in Eastern India with a sequence divergence greater than 4% from subgenotypes D1–D4 [26]. Phylogenetic analysis based on the partial HBV genome sequences of two HBV/D isolates from Papua, Indonesia, provided initial evidence for subgenotype D6 [27], which was later verified in 2009 using three complete HBV genome sequences [28]. Subgenotype D7 was identified by full-length genome analysis of HBV/D strains from Tunisian blood donors, which demonstrated clear phylogenetic separation, genomic divergence of over 4% from all other HBV/D subgenotypes, and relatedness to HBV/E in the precore/core gene [29]. Four complete HBV genomic sequences identified in Niger in 2010 clustered on a distinct branch within HBV/D and were classified as a novel subgenotype D8, despite evidence of HBV/D and E intergenotypic recombination [30]. Other recombinant strains, in this case, HBV/D and C recombinants identified in Eastern India, were assigned to a new subgenotype D9 [31]. In 2017, subgenotype D10 was identified in Ethiopia based on analysis of fifteen HBV/D complete genomes that showed a novel distinct cluster supported by a high bootstrap value and >4% nucleotide divergence from other known subgenotypes [32]. Subgenotype D11 was distinguished in two serum samples collected in 2011 and 2018 from a single individual living in Napo County, China [33].

Despite the establishment of guidelines for HBV subgenotyping, a number of misclassifications have been reported and new classifications proposed, some involving HBV/D subgenotypes [12,13,24,34,35,36]. Yousif and Kramvis (2013) showed a low intergroup divergence of 2.6 ± 0.6 (% ± SD) and low bootstrap support of 56% for separate clustering of D3 and D6 and significant high bootstrap support of 99% for co-clustering of D3 and D6, suggesting that D6 is not a separate subgenotype, but rather a clade of subgenotype D3. Additionally, the group proposed that subgenotype D8 should be considered a recombinant genotype D/E rather than HBV/D subgenotype [24]. Similarly, subgenotype D9 (D/C recombinant) was also considered misclassified [12]. With regard to D1 and D2, there seems to be a consensus in the acceptance of true subgenotypes despite intergroup divergence being below the threshold of 4% used to separate subgenotypes. The justification for classifying D1 and D2 as subgenotypes is based upon well-resolved phylogenetic clustering, supported by high bootstrap values, different serological subtypes, and distinct geographic distributions [24]. In 2010, Pourkarim et al. [34] coined the term “quasi-subgenotype” to address the misclassification of HBV subgenotypes lacking sufficient nucleotide divergence and bootstrapping value for classification as subgenotypes. At least three reports of quasi-subgenotypes for HBV/D are documented in the literature (Figure 1). The first involved four strains from Taiwanese aborigines that were grouped into a new cluster supported by a branch with 99% bootstrap value and 3.4–5.8% nucleotide divergence over the entire genome from other known HBV/D subgenotypes. Since the divergence with D2 was the lowest (3.4 ± 0.2), this lineage was designated as the novel quasi-subgenotype D2 [37]. In 2020, a novel quasi-subgenotype D12 with inter-group nucleotide divergence of <4% in relation to both D1 and D2 subgenotypes was identified in two patients from Ghana residing in Belgium [38]. Finally, based on the analysis of the HBV strains circulating in individuals from settlements in Western Greenland, Schneider and colleagues (2021) identified a novel HBV/D quasi-subgenotype with a nucleotide distance separation of ~3% from D1 and D2 [39].

### 2.2. Geographic Distribution of HBV/D Subgenotypes

HBV/D is prevalent worldwide, but most commonly identified in Southeastern Europe, the Mediterranean Basin, Middle East, and the Indian subcontinent [24]. HBV/D accounts for nearly all HBV cases in Albania, Greece, Italy, Iran, Lebanon, Serbia, and Turkey [40].

**Figure 2:**
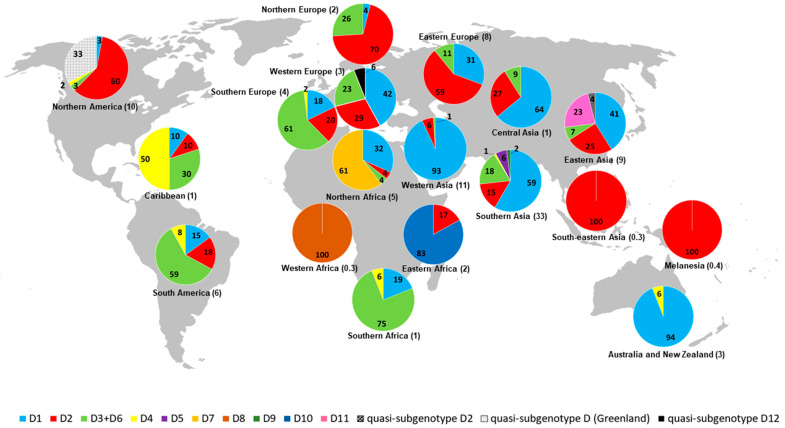
Geographic distribution of HBV/D subgenotypes based on 1156 complete genome sequences available in GenBank. Numbers in pie charts represent the percentage values of the frequencies of HBV/D subgenotypes in each region. Numbers in parentheses represent the percentage values of the total number of HBV/D sequences in each region. The map was reconstructed using Wikimedia Commons (https://commons.wikimedia.org/wiki/File:BlankMap-World-noborders.png) (accessed on 19 April 2023). This figure is similar but not identical to the original image and therefore for illustrative purposes only.

Table 1 shows the frequencies of HBV/D subgenotypes by country and geographic region based on 1156 HBV/D complete genome sequences available in GenBank. Data on the accession numbers, subgenotypes, and geographic regions of the sequences used in this study are presented in Table S1. Worldwide frequencies of HBV/D subgenotypes are as follows: D1, 44.3%; D2, 24.2%; D3 + D6, 16.9%; D4, 1.8%; D5, 1.8%; D7, 2.9%; D8, 0.3%; D9, 0.5%; D10, 1.3%; D11, 2.0%; quasi-subgenotypes (all), 3.9%. Although D1 is almost twice as prevalent as D2 (44.3% vs. 24.2%), the latter is distributed in more geographic regions (15 vs. 14). Overall, subgenotypes D1–D4 show a more widespread global distribution, while D5–D11 and quasi-subgenotypes are restricted to one or two countries within the same geographic region. D1 is more frequently detected in Southern Asia (43.9%), D2 in Northern America (25.4%), D3 + D6 in Southern Asia (34.4%), D4 in South America (28.6%), D5 in Southern Asia (100%), D7 in Northern Africa (100%), D8 in Western Africa (100%), D9 in Southern Asia (100%), D10 in Eastern Africa (100%), and D11 in Eastern Asia (100%) (Table 1). Southern Asia is the geographic region with the highest number of HBV/D complete genome sequences (378/1156, 32.7%) and subgenotypes/quasi-subgenotypes (6/13), followed by Eastern Asia and Northern America (5/13), the Caribbean, Northern Africa, South America, Southern Europe, and Western Europe (4/13), Central Asia, Eastern Europe, Northern Europe, Southern Africa, and Western Asia (3/13), Australia, New Zealand, and Eastern Africa (2/13), and Melanesia, Southeastern Asia, and Western Africa (1/13). Complete genome sequences of HBV/D have not been identified in Central America, Middle Africa, and Polynesia (Figure 2).

### 2.3. Origin and Worldwide Spread of HBV/D

The temporal and geographic origins of HBV in humans as well as major routes of transmission in the past remain a topic of debate [41,42,43]. Considering its global prevalence and existence of comparable viruses in other animals, including non-human primates, it is widely assumed that HBV has been endemic in human populations for thousands of years. Indeed, recent findings of HBV DNA from archaeological human remains, some date as far back as ~10,500 years, provide new perspectives on the evolutionary history of HBV [19,21,22,44,45]. Unexpectedly, phylogenetic analysis of HBV sequenced from a Korean mummy from the 16th century revealed a close relationship with modern HBV/C sequences [44]. Likewise, phylogenetic analysis of HBV retrieved from an Italian mummy dated from the mid-sixteenth century showed commonality within the diversity of modern HBV strains (HBV/D) [45]. In addition, an analysis of 12 ancient HBV genomes, dated between 800 and 4500 years ago, led to them being grouped either within or in a sister relationship with extant human or other ape HBV clades, with genome properties similar to those of modern HBV [22]. Such phylogenetic patterns suggest a long-term association of the modern HBV genotypes with the human population. Krause-Kyora et al. (2018) shed light on the timescale of infection in an analysis of three ancient (two Neolithic and one medieval) HBV genomes recovered from human skeletons in Germany [21]. Strikingly, the Neolithic HBV genomes (~7000 and ~5000 years ago) were not grouped with any human-associated HBV sequences, appearing to represent distinct lineages with no close relatives today that possibly went extinct. The two Neolithic strains were most closely related to those infecting African non-human primates, suggesting reciprocal cross-species transmission at one or possibly several times in the past. By contrast, the genome from the 1000-year-old isolate clustered with the modern D4 subgenotype [21]. Further information was uncovered by Kocher and coworkers (2021), who generated HBV genomic data from 137 Eurasians and Native Americans dated from ~10,500 to ~400 years ago. Their findings showed that the HBV lineage that prevailed throughout western Eurasia for ~4000 years, designated Western-Eurasian Neolithic-to-Bronze Age (WENBA), declined around the end of the second millennium before the current era (BCE) and was almost completely replaced (HBV/G being the only remnant of this prehistoric HBV diversity) by the modern HBV strains [19]. Considering these findings, it is suggested that the current diversity of genotypes only reflects a relatively recent part of the phylogeographic history of HBV, challenging the notion that the current viral diversity reflects early human dispersals out of Africa.

Owing to the lack of agreement concerning the HBV substitution rate, the times of origin and divergence of HBV (sub)genotypes are largely uncertain. Studies using external calibrations based on human migrations or including ancient sequences have estimated slower rates (long-term), while rates estimated using tip-dating analyses of modern HBV sequences tend to be faster (short-term), leading to a wide range of estimated substitution rates (7.72 × 10^−4^–3.7 × 10^−6^ substitutions per site per year) [42,46,47]. Time scale reconstructions based on long-term evolutionary rates have recovered a significantly older time of emergence of the most recent common ancestor (tMRCA) than those based on short-term rates. Therefore, tip-dating analyses of modern heterochronous HBV sequences may be more appropriate for dating recent dispersal events, whereas the use of deep calibrations is more effective to estimate events distant in time (e.g., origin of viral genotypes) [42,48]. Consequently, previous studies on the temporal dynamics of HBV/D and its subgenotypes have reported widely differing estimates [20,48,49,50,51,52,53,54].

The first occurrence of HBV/D isolates dates back to the late Iron Age (~2800 years ago), with the majority of reported cases detected in Central Asia [19,22]. These ancient genomes appeared phylogenetically basal to most European medieval isolates, supporting an Asian origin of HBV/D, followed by east-to-west expansion [19,22]. In addition, several HBV/D genomes from medieval Europe were basal to subgenotypes that currently prevail in other continents, such as D3 and D4, indicating that European colonization and modern migrations may have driven the spread of these lineages [19]. Interestingly, this theory is consistent with previous spatiotemporal reconstruction analyses that demonstrated a substantial role of Europe in the exportation of subgenotypes D2 (Estonia, Poland, Russia, and Serbia), D3 (Italy and Spain), and D4 (Portugal) to countries from the Americas, probably during mass migratory movements in the 19th and 20th centuries [48,55]. Within that same period, migration events from Western Asia, a plausible place of origin of D1 [48,52], were related to the introduction of this subgenotype in South American and Caribbean countries [48]. Another finding of interest was that an ancient HBV (HBV-DA27) found in Kazakhstan (dated 1600 years ago) fell basal to the modern sequences of subgenotype D5 [22], which today circulates mainly in India and is the sole subgenotype detected in the isolated primitive Paharia ethnic community of Eastern India [56]. Indeed, DA27 and the Paharia people are linked by their East Asian ancestry [22].

The global dispersal patterns of HBV/D have been phylogenetically defined into regional clusters at different levels depending on the geographic origin of sampling [20]. Interestingly, high levels of regional clustering observed in Greenland (100%, D2), New Zealand (97%, D1), Japan (83%, D2), Tunisia (66%, D7), and China (65%, D1) as well as significant viral genetic diversity suggest that HBV/D dispersal in these regions is the result of onward transmissions of a single or few strains introduced in the populations in the past. Conversely, low levels of monophyly clustering in Iran, Syria, Turkey, Belgium, India, Lebanon, and Russia suggest significant population mobility giving rise to infections in these regions [20].

### 2.4. Clinical Associations of HBV/D Subgenotypes with Infection

The constant evolution of HBV has led to continued selection of variants in response to pressures exerted by the host’s immune system, antiviral therapy, and vaccination, resulting in the emergence of mutations with important implications for prevention, diagnosis, treatment, and prognosis of infection. Accumulating evidence suggests that (sub)genotypes influence natural history, clinical outcomes, and treatment response of HBV [15,16,17,18]. One potential underlying reason is that HBV mutations are significantly more prevalent in some (sub)genotypes relative to others [14,57].

Data from a number of country-specific studies suggest that HBV/D has a more aggressive disease course than HBV/A, and HBV/C is linked to more severe liver disease than HBV/B [18,58,59]. However, research on the clinical significance of HBV subgenotypes is still scarce. Pre-S deletions, C1653T in enhancer II, and T1753V and A1762T/G1764A in the basal core promoter have been shown to be significantly associated with an increased risk of HCC compared to cases of HBV without mutations [60]. Large-scale analysis of 6479 HBV genome sequences from genotypes A to H has provided comprehensive insights into the relationships of the most clinically relevant mutations with HBV genotypes and subgenotypes [14]. Remarkably, significant differences in the frequencies of HCC-associated mutations among HBV genotypes were observed. HBV/G showed the highest frequency (97.5%) to a significant extent, followed by HBV/C (49.7%) and HBV/D (30.9%). Individually, the frequencies of A1762T/G1764A, T1753V, and C1653T in HBV/D were 21.5%, 16.6%, and 8.8%, respectively, while pre-S deletions were not detected. Additionally, significant differences in the frequencies of HCC-associated mutations were observed among HBV/D subgenotypes (D1–D6), with D1 displaying the highest rate (36.4%) [14]. Interestingly, a report from India showed a marked association of subgenotype D1 with chronic liver disease with elevated ALT as well as a higher HBsAg positivity rate compared with D2, D3, and D5 [61]. Moreover, Indian patients infected with D1 were more susceptible to severe liver damage followed by rapid progression to HCC than those infected with D3. Results from complementary in vitro assays support epidemiological data, revealing that D1 replicates faster and triggers more ER stress compared to D3 [62]. Similarly, D1 was independently shown to be associated with a higher risk of HCC than D2 and D3 [63] (Table 2).

Regarding the influence of (sub)genotypes on the response to antiviral treatment, several studies suggest that HBV/D is the least sensitive to interferon (IFN) [17,43,64,65]. In particular, a multicenter study of HBeAg-positive patients treated with peg-IFN-α showed rates of HBeAg clearance for HBV/A-D of 47%, 44%, 28%, and 25% (*p* = 0.01), respectively, indicating that HBV/D has poorer response to peg-IFN-α therapy than the other three common genotypes [65]. Given these findings, HBV genotyping can be useful in patients being considered for peg-IFN therapy [3]. By contrast, the link between (sub)genotype and the response to treatment with NA-based regimens remains unclear. Despite the efficacy of NA therapy in sustained viral suppression, long-term use of treatments with a low barrier to resistance, such as lamivudine (LAM) and adefovir (ADV), can contribute to the emergence of resistant HBV mutants. Therefore, entecavir (ETV), tenofovir disoproxil fumarate (TDF), and tenofovir alafenamide (TAF) have been recommended as first-line monotherapy for chronic hepatitis B [3]. Data from an earlier meta-analysis revealed no significant association between HBV/A-D and the response to NAs [66]. However, results from a nine-year longitudinal study demonstrated increased HBsAg clearance rates during NA therapy in patients infected with HBV/A compared to those with HBV/C [67]. Comparative analysis of HBV/A and HBV/D showed that patients infected with HBV/D achieved a higher rate of a sustained virologic response to LAM therapy than the HBV/A-infected group (28.8% and 3.5%, respectively, *p* = 0.0359) [68]. Khatun et al. (2022) reported that within the HBV/D subgenotypes, D1/D2 showed greater susceptibility to TDF/ETV while D3/D5 exhibited a poorer response in vitro. Moreover, the HBV load was significantly decreased in TDF-treated patients carrying D1/D2 but only marginally reduced in D3/D5-infected patients. Surprisingly, the replacement of signature residues in D3/D5 HBV clones with the corresponding amino acids in D1/D2 improved susceptibility to TDF/ETV [69]. These findings are in agreement with previous reports that HBV/D1 is more sensitive to ETV therapy than HBV/D3 in vitro [62]. Significant differences among HBV (sub)genotypes in terms of frequency of antiviral-resistant mutations have been reported [14]. The frequency of LAM-resistant mutants in HBV/D was 7.4% (ranging from 32.5% in HBV/G to 0.3% in HBV/E), whereas the rate of ADV-resistant mutants was 0.1% (ranging from 0.9% in HBV/C to 0 in HBV/E-H), and ETV resistance was 0.2% (ranging from 0.4% in HBV/C to 0 in HBV/A, B, E–G). Mutants with reduced TDF susceptibility were only observed in HBV/B and C (0.1% for both). Among the HBV/D subgenotypes (D1–D6), the frequency of LAM-resistant mutants was highest in D2 (16.2%) [14] (Table 3).

HBsAg contains a highly conserved antibody-neutralizing epitope cluster known as the “a” determinant, which spans amino acids 124–147. Mutations in or around this region can cause conformational changes, which might impact the binding of neutralizing antibodies generated during natural infection or following active or passive immunization [70]. These immune escape mutations account for a number of possible effects, such as evasion of anti-HBV immunoglobulin therapy and vaccine-induced immunity as well as false-negative results by commercial HBsAg assays (“false” occult hepatitis B) [71,72]. Notably, significant differences in the frequencies of escape mutations among HBV genotypes have been observed, with HBV/A-D and G showing higher rates than E, F, and H. In particular, the frequency of escape mutations in HBV/D was 9.3% (ranging from 14.7% in HBV/B to 0 in HBV/H), with subgenotype D4 displaying the highest rate (19%) within HBV/D [14]. Similar findings showed that HBV/A-D harbor more escape mutations than the other genotypes [73]. Of note, Di Lello and colleagues [74] found a higher prevalence of diagnostic failure mutations in patients infected with HBV/D (33.3%) than those infected with HBV/A (17.4%) or HBV/F (2.3%) (*p* < 0.001). Moreover, lower levels of HBsAg secretion for HBV/D compared to other genotypes were observed in several in vitro studies [75,76,77], as well as in the serum of infected patients [78,79]. Interestingly, the characteristic 33 nt deletion of HBV/D has been associated with the reduction in HBsAg secretion [80]. The above findings point to a possible association between HBV/D and the occurrence of “false” occult HBV infection.

## 3. Conclusions

HBV/D has unique molecular and epidemiological characteristics and is the most widely distributed genotype worldwide. Considering the introduction of increasing numbers of new viral subgenotypes, we have provided an up-to-date review of the history of HBV/D subgenotyping and misclassifications in this study. Based on analysis of over 1000 HBV/D complete genome sequences, we illustrated the distinct geographic distributions of subgenotypes revealing their prevalence and areas of high and low genetic variability. Recent discoveries on HBV ancestral strains that have prevailed for ~4000 years throughout western Eurasia shed light on new perspectives regarding the origins of all known modern HBV strains. Large-scale paleogenomic analyses facilitated the detection of HBV/D genomes dating back to the late Iron Age, suggesting an Asian origin for this genotype followed by east-to-west expansion. Spatiotemporal reconstruction analyses uncovered the differential distribution patterns of HBV/D subgenotypes worldwide and the role of mass migratory movements in virus spread, such as those occurring during the 19th and 20th centuries from Europe to the Americas. The heterogeneous prevalence of clinically relevant mutations as well as distinct disease outcomes and responses to antiviral therapy among HBV/D subgenotypes highlight the complexity of this genotype and further support the importance of HBV subgenotyping in the management and treatment of chronic hepatitis B.

## Figures and Tables

**Table 1:** Distribution of HBV/D subgenotypes by country and geographic region.

Genotype D (*n* = 1156) ^1^		
Subgenotype (%)	Country (%)	Region (%) ^2^
D1 (44.3)	Iran (25.2) India (17.6) Turkey (11.5) China (6.6) New Zealand (6.6) Syria (5.9) Lebanon (5.3) Russia (4.9) Tunisia (2.7) Belgium (2.1) Argentina (1.8) Mongolia (1.2) Italy (1.0) Pakistan (1.0) Uzbekistan (1.0) Egypt (0.8) South Africa (0.6) USA (0.6) Belarus (0.4) Greece (0.4) Japan (0.4) Poland (0.4) Bangladesh (0.2) Brazil (0.2) Cuba (0.2) France (0.2) Germany (0.2) Kazakhstan (0.2) Latvia (0.2) Serbia (0.2) Spain (0.2) Tajikistan (0.2) Venezuela (0.2)	Southern Asia (43.9) Western Asia (22.7) Eastern Asia (8.2) Australia and New Zealand (6.6) Eastern Europe (5.7) Northern Africa (3.5) Western Europe (2.5) South America (2.1) Southern Europe (1.8) Central Asia (1.4) Northern America (0.6) Southern Africa (0.6) Caribbean (0.2) Northern Europe (0.2)
D2 (24.2)	USA (25) Russia (16.8) India (16.4) Japan (9.3) Brazil (3.6) Estonia (3.6) Belgium (2.5) Spain (2.5) Bangladesh (2.1) Latvia (1.8) Lebanon (1.8) New Caledonia (1.8) Poland (1.8) Argentina (1.1) Belarus (1.1) Ethiopia (1.1) Iran (1.1) Malaysia (1.1) Germany (0.7) Greece (0.7) Tajikistan (0.7) Turkey (0.7) Sudan (0.7) Cuba (0.4) Greenland (0.4) Kazakhstan (0.4) Serbia (0.4) Sweden (0.4) Syria (0.4)	Northern America (25.4) Southern Asia (19.6) Eastern Europe (19.6) Eastern Asia (9.3) Northern Europe (5.7) South America (4.6) Southern Europe (3.6) Western Europe (3.2) Western Asia (2.9) Melanesia (1.8) Central Asia (1.1) Eastern Africa (1.1) Southeastern Asia (1.1) Northern Africa (0.7) Caribbean (0.4)
D3 + D6 (16.9)	India (34.4) Brazil (16.4) Italy (13.8) Argentina (6.2) Russia (3.6) Botswana (3.1) China (3.1) South Africa (3.1) Sweden (2.6) Belgium (2.1) Belarus (1.5) Canada (1.5) Serbia (1.5) France (1.0) Haiti (1.0) Sudan (1.0) Cuba (0.5) Estonia (0.5) Germany (0.5) Kazakhstan (0.5) Mongolia (0.5) Spain (0.5) Syria (0.5) USA (0.5)	Southern Asia (34.4) South America (22.6) Southern Europe (15.9) Southern Africa (6.2) Eastern Europe (5.1) Eastern Asia (3.6) Western Europe (3.6) Northern Europe (3.1) Northern America (2.1) Caribbean (1.5) Northern Africa (1.0) Central Asia (0.5) Western Asia (0.5)
D4 (1.8)	Brazil (28.6) India (19.0) Cuba (14.3) Canada (9.5) Haiti (9.5) Australia (4.8) New Zealand (4.8) South Africa (4.8) Spain (4.8)	South America (28.6) Caribbean (23.8) Southern Asia (19.0) Australia and New Zealand (9.5) Northern America (9.5) Southern Africa (4.8) Southern Europe (4.8)
D5 (1.8)	India (95.2) Bangladesh (4.8)	Southern Asia (100)
D7 (2.9)	Tunisia (100)	Northern Africa (100)
D8 (0.3)	Niger (100)	Western Africa (100)
D9 (0.5)	India (100)	Southern Asia (100)
D10 (1.3)	Ethiopia (100)	Eastern Africa (100)
D11 (2.0)	China (100)	Eastern Asia (100)
quasi-subgenotype D12 (0.2)	Belgium (100)	Western Europe (100)
quasi-subgenotype D2 (0.3)	China (100)	Eastern Asia (100)
quasi-subgenotype D (3.4)	Greenland (100)	Northern America (100)

^1^ Based on 1156 HBV/D complete genome sequences available in GenBank. ^2^ Sequences were grouped into 21 geographic subregions according to the UN Statistics Division (https://unstats.un.org/unsd/methodology/m49/#geo-regions) (accessed on 19 April 2023).

**Table 2:** Associations of HBV/D subgenotypes with liver disease.

HBV/D Subgenotype Association	Reference
D1 has a significantly higher rate of HCC-associated mutations than D2–D6.	[14]
D1 is more significantly associated with chronic liver disease with an elevated ALT compared with D2, D3, and D5.	[61]
D1 is more significantly associated with a higher HBsAg positivity rate than D2, D3, and D5.	[61]
D1 infection is associated with a higher susceptibility to severe liver damage and rapid progression to HCC than D3.	[62]
D1 replicates faster and triggers more ER stress than D3 in vitro.	[62]
D1 is associated with a higher risk of HCC than D2 and D3.	[63]

**Table 3:** Associations of HBV/D subgenotypes with response to antiviral therapy.

HBV/D Subgenotype Association	Reference
D1/D2 have a greater susceptibility to TDF/ETV than D3/D5 in vitro.	[69]
D1/D2 viral loads are lower than D3/D5 in TDF-treated patients.	[69]
D1 has a greater susceptibility to ETV than D3 in vitro.	[62]
D2 has a significantly higher rate of LAM-resistant mutants than D1–D6.	[14]

## Data Availability

Not applicable.

## Supplementary Materials

The following supporting information can be downloaded at: https://www.mdpi.com/2076-2607/11/5/1101/s1, Table S1: Dataset of HBV/D complete genome sequences.
